# Unstable Total Hip Arthroplasty: Should It Be Revised Using Dual Mobility Implants? A Retrospective Analysis from the R.I.P.O. Registry

**DOI:** 10.3390/jcm12020440

**Published:** 2023-01-05

**Authors:** Alberto Di Martino, Matteo Brunello, Barbara Bordini, Valentino Rossomando, Leonardo Tassinari, Claudio D’Agostino, Federico Ruta, Cesare Faldini

**Affiliations:** 1 I Orthopedic and Traumatology Department, IRCCS Istituto Ortopedico Rizzoli, 40136 Bologna, Italy; 2 Department of Biomedical and Neurimotor Science-DIBINEM, University of Bologna, 40136 Bologna, Italy; 3 Medical Technology Laboratory, IRCCS Istituto Ortopedico Rizzoli, 40136 Bologna, Italy

**Keywords:** dual mobility, dislocation, failure, registry, revision, total hip arthroplasty, tribology

## Abstract

Total hip arthroplasty (THA) is one of the most common surgical procedures in orthopedics; however, it is subjected to different kinds of failures, one of them being dislocation. Many different prosthetic designs have been developed to overcome this problem, such as dual mobility coupling. The main purpose of this article is to determine whether there are differences regarding the revision surgery of unstable THA comparing the risk of failure between dual mobility cup (DMC) implants, standard implants, and among different head sizes. A registry-based population study has been conducted by analyzing data collected by the Emilia Romagna Registry of Orthopedic Prosthetic Implants (RIPO), including a total of 253 implants failed for dislocation and instability that were operated on by cup revision surgery between 2000 and 2019. The selected population has been divided into two groups based on the insert type: standard and DMC. The age at revision surgery was significantly lower in the standard cup group with respect to DMC (*p* = 0.014 *t*-test), with an average age of 71.2 years (33–96 years range) for the standard cups and 74.8 years (48–92 years range) for the DMC group. The cumulative survival of DMC implants was 82.0% at 5-years, decreasing to 77.5% at a 10-year follow-up, which is not significantly different from standard cups (*p* = 0.676, Log-Rank test). DMC implants showed a significantly lower risk of re-revision for dislocation compared to standard cups (*p* = 0.049). Femoral heads ≥36 mm had a higher overall survival compared to smaller femoral heads (*p* = 0.030). This study demonstrated that DMC or femoral heads ≥36 mm are a valid choice to manage THA instability and to reduce the revision rate for dislocation at a mid-term follow-up; in those selected and targeted patients, these options should be taken into consideration because they are associated with better outcomes.

## 1. Introduction

Total hip arthroplasty (THA) is one of the most common surgical procedures in orthopedics. It reduces pain and gives back functionality in patients with hip disease, such as hip osteoarthritis. However, it is subject to different kinds of failures. With the constant advancement in life expectancy, the numbers of prosthetic failures have increased [1]. The need for revision surgery in THA has grown in the last decades [2]. The most common causes of revision are aseptic loosening, instability (defined as recurrent dislocations), septic loosening, and periprosthetic fractures. The dislocation of the implant represents a frequent complication [3] leading to a primary implant failure, and patients with an implant failure due to dislocation show a rate of repeated dislocation from 18 to 33% [4,5,6,7]. Furthermore, in addition to being a strong predictor for patient dissatisfaction, it is a cost to health systems [8]. In recent history, many different prosthetic designs have been developed to overcome the risk of dislocation, including larger femoral heads, constrained liners, elevated-rim acetabular liner, and dual-mobility articulations. Particularly interesting is the dual mobility cup, as it combines the decrease in probability of dislocation, given by a greater jumping distance, with the small friction produced by the small head [9]. This concept was proposed and then developed in 1976 by Bousquet in France, who is assumed as the designer of the “double mobility cup” (DMC). 

Currently, the most common indication for dual mobility is primary surgery revision following implant instability [10]. Many surgeons started to extend surgical indications in patients undergoing primary implants when there is higher risk of dislocation, for instance: Parkinson’s disease, older people with poor soft tissues quality, proximal femoral replacement surgery, patients undergoing spine fusion surgery, and not compliant or mentally disabled patients [11,12,13,14]. In these, total hip arthroplasty is widely regarded as a viable treatment strategy; however, standard implants may result in recurrent dislocation and instability due to muscular insufficiency. Therefore, the use of DMC in revision surgery began rising [15,16]. In the literature, there are several registry articles analyzing the outcomes of DMC implants in revision surgery, aimed at the reduction of the risk of dislocation, but only short-term follow up studies are available so far [17,18,19]. The main purpose of this registry study was to determine, with a follow-up of 10 years, whether there are differences in terms of the survival of revision surgery implants for THA instability, comparing the risk of failure between DMC implants and standard prosthetic implants; moreover, the contribution of different femoral head sizes to implant survival in THA revision surgery performed for instability and the causes of failure of DMC compared to standard implants were analyzed.

## 2. Materials and Methods

A registry-based population study has been conducted by reporting and analyzing data collected by the Emilia Romagna Registry of Orthopedic Prosthetic Implants (called RIPO for Registro dell’Implantologia Protesica Ortopedica). Emilia Romagna (ER) is an Italian region with 4.5 million inhabitants, and report data about hip, knee, and shoulder arthroplasty procedures performed in the region are collected in the RIPO Registry. Founded in 1990, RIPO has a capture rate of approximately 98% on the implants performed in all the orthopedic departments of the region involving a total of 62 hospitals (both public and private). The specific design of this register allows comparisons with other important national registries to be made. 

For the study, we selected the revision of THA performed in the period between 2000 and 2019. Implant failures were considered and reported up to the 31 December 2020. Data extraction from the database was made on 8 August 2022. Ethical approval for the study was not necessary because the registry collects data as standard practice on all patients, using a format protecting their identity. A total of 93.521 total hip arthroplasties were performed in ER during the selected period, collecting 4.632 implant failures requiring revision. Procedures performed on patients living outside the ER region were excluded, to minimize bias due to loss at follow-up. 

**Figure 1:**
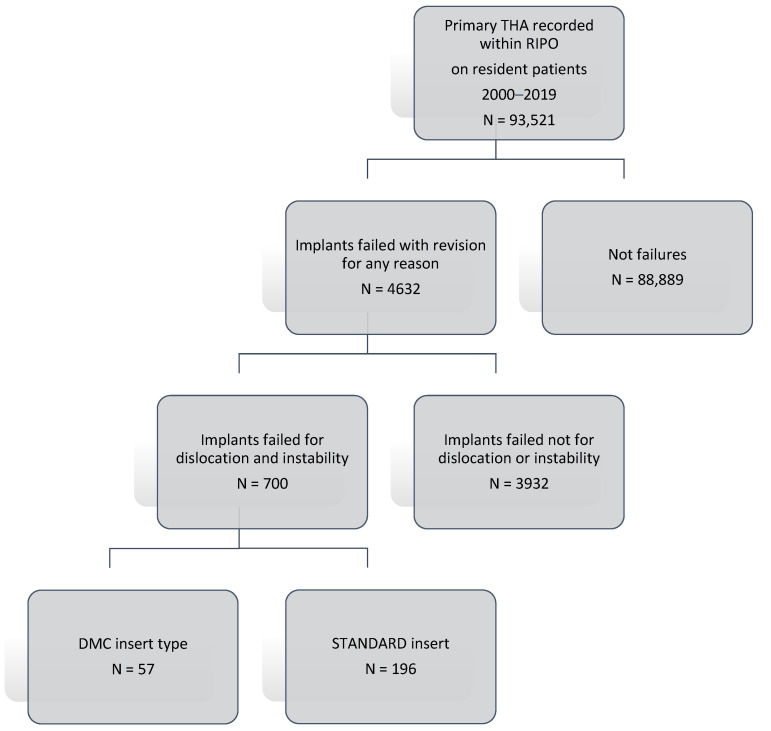
Patient selection flow-chart.

A total of 700 implants that failed for dislocation and instability which required revision surgery were collected (Figure 1); of those, 253 implants with only cup revision performed in 19 years were included in the study. Revisions were divided for the implants: DMC and standard cup. The following data were considered: sex, age at surgery, BMI, femoral head size for standard insert, dual mobility insert, revisions implant survival rates at 5- and 10-years of follow-up. The selected population was divided into two groups based on insert type: standard and DMC, with 196 and 57 implants, respectively. During the period 2000–2019, a total of 57 DMC were used in revision THAs, the most used being Trident PSL HA Cluster (n = 8; 14.0%), SAINT-GENIX FH ORTHOPEDICS (n = 6; 10.5%), POLARCUP ORTHO-ID (n = 4; 7.0%), AVANTAGE 3P BIOMET (n = 4; 7.0%), TRIDENT PSL HA CLUSTER STRYKER ORTHOPAEDICS (n = 3; 5.3%), AVANTAGE CEMENTED BIOMET (n = 3; 5.3%), and DMX TRANSYSTEME (n = 3; 5.3%); other implants were used in two or less patients each (n = 24; 42.1%). Standard implants where the femoral head was revised were divided according to the femoral head size: ≤28 mm, 32 mm, and ≥36 mm.

Descriptive statistics were used to summarize the data, presented as the median and mean with standard deviation (SD) for continuous variables and as the frequency with percentage (%) for categorical variables. The statistical significance was calculated using the chi-square test for qualitative data and t-test for continuous data. A *p*-value of  <0.05 was considered statistically significant. Kaplan–Meier survivorship analysis was performed using the revision of any component as the endpoint and survival times of unrevised THAs taken as the last date of observation (31 December 2020, or date of death). The log-rank test was used to compare survivorship between the two groups. Statistical analyses were performed using SPSS 14.0, version 14.0.1 (SPSS Inc., Chicago, IL, USA) and JMP, version 12.0.1 (SAS Institute Inc., Cary, NC, USA, 1989–2007).

## 3. Results

The analysis of the population started from the description of demographics and surgical information. Of the 253 implants, 166 were females (65.6%) and 87 males (34.4%), sex is equally distributed in the groups (*p* = 0.163 Pearson’s chi-squared test) and a prevalence for each group of an age range between 70–79 years (Table 1).

Population was divided by Body Mass Index (BMI). There was a prevalence of the overweight condition, with 108 patients (42.7%), followed by normal weight, with 61 patients (24.1%), and obese with 42 (16.6%) (Table 2). 

The analysis was performed for the two groups involved in the study, with DMC and standard cup in revision, respectively, of 57 (22.5%) and 196 (77.5%) implants. No Statistically significant differences were identified for gender between the standard and DMC groups (*p* = 0.163 Pearson’s chi-squared test). The average age was 71.2 years (range 33–96 years) for the standard cup and 74.8 years (range 48–92 years) for the DMC group. The age at revision surgery was significantly lower in patients operated with standard cups compared to DMC implants (*p* = 0.014 *t*-test). No differences were observed in terms of BMI between the standard and DMC groups (Table 3) (*p* = 0.488 Pearson’s chi-squared test).

**Figure 2:**
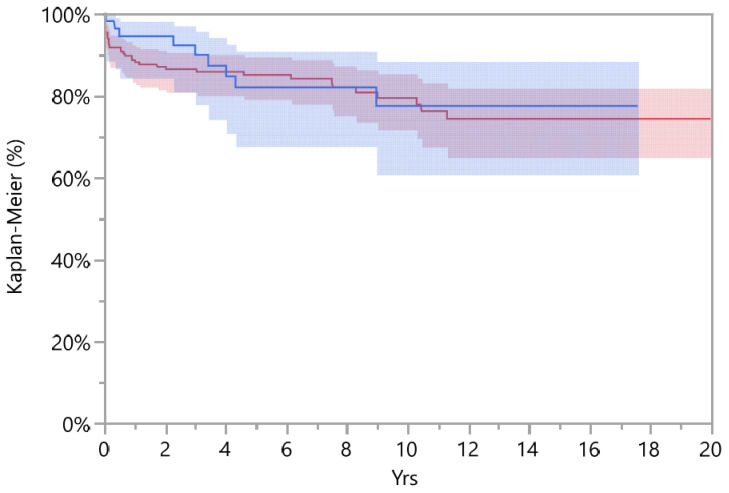
Survival standard (red line and area) and DMC implant (blue line and area) with Kaplan–Meier (%) diagram.

The cumulative survival of DMC implants was 82.0% (CI 67.7–90.9) at 5-years, decreasing to 77.5% (CI 60.8–88.4) at the 10-year follow-up; when compared to standard cups, the latter did not show a significant difference (*p* = 0.676, Log-Rank test), and showed an overall survival at the 5- and 10-year follow-up of 85.1% (CI 79.2–89.5) and 77.9% (CI 69.7–84.3), respectively (Figure 2). All data are reported in Table 4.

In the study, all causes of re-failure for both groups during the follow-up period were collected, with 36 failed implants in the standard cup group (36/196 18.3%) and 9 in the DMC group (9/57 15.7%). Dislocation was the leading cause of revision in the standard group with 11 cases, while in the DMC group, aseptic cup loosening was the main cause of failure with 3 cases. All the causes of re-revision are collected in Table 5.

Comparing the risk of re-revision for dislocation in the follow-up period, 11 standard cup implants failed for dislocation, while no DMC implants have failed for the same reason, and DMC showed a significantly lower risk of re-revision for dislocation compared to standard cups (*p* = 0.049, Log-Rank test). 

**Figure 3:**
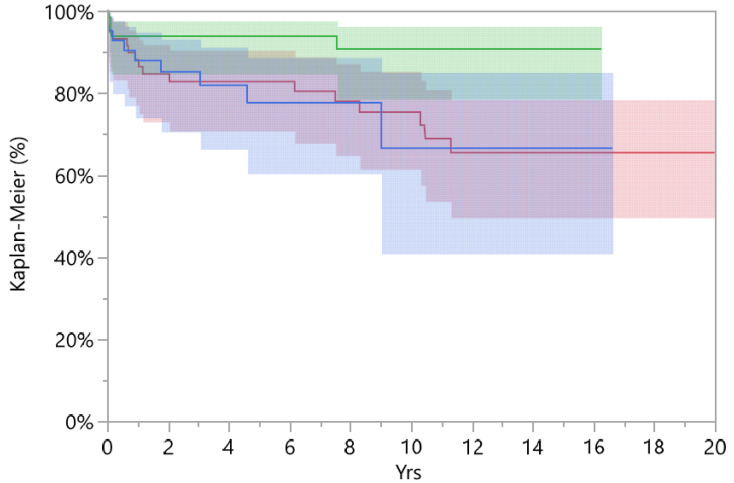
Survival implants divided to femoral head size: red line ≤ 28 mm, blue line = 32 mm, and green line ≥ 36 mm.

The standard cup group was investigated for the femoral head size used in revision surgery; from 196 implants, 171 had a revised femoral head. Femoral heads were divided according to the size: ≤28 mm, 32 mm, and ≥36 mm. The cumulative survival at 15-years for a femoral head diameter of ≥36 mm was 90.7% (CI 78.6–96.3), 32 mm reached 66.5% (CI 40.8–85.1), and survival in implants with femoral heads of ≤32 mm was 65.4% (CI 49.6–78.4) (Figure 3).

A femoral head diameter of ≥ 36 mm had higher overall survival compared to smaller femoral heads (*p* = 0.030, Log-Rank test). In our study, there was no dislocation for DMC, 2 for heads of ≥36 mm, 4 in the 32 mm group, and 5 in the 28 mm group. No difference in survival comparing a femoral head diameter of ≥36 mm and DMC was found (*p* = 0.18, Log-Rank test).

## 4. Discussion

The most important finding of the present study was that DMC demonstrated a lower risk of re-revision for dislocation compared to standard cups at a 10-year follow-up. Patients operated with standard cups were younger at revision surgery compared to patients with DMCs, averaging 71.2 vs. 74.8 years, respectively. A femoral head with a diameter of ≥36 mm showed a better survival rate for instability compared to smaller heads, but was not different from DM.

The main limit of the current study, intrinsic to the use of a registry, is that the design is retrospective, and data came from observation [20]; therefore, it can only provide information about the associations between variables, and these may or may not arise from causality. Moreover, patients who performed THA re-revision in other districts were lost at follow-up and were not registered. 

We found that DMC was used more frequently in revision surgery for instability in patients slightly older than those receiving a standard cup, [17,21,22], confirming the findings of Mohaddes et al. which showed an average age of 75 years for DMC implants, and 73 years for standard cup. Viste et al. [23], in a retrospective study of DMC use in hip revision surgery in 383 patients, reported a mean age at surgery of 78 years. The tendency in the literature and the findings in our study seem to emphasize the use of DMC in the elderly population, also highlighting that revisions are much more common in this age group. Several studies showed that obesity could be related to increased peri operative complications [24,25] and risk of failure because of the mechanical stress applied on the implant surfaces [26]. Unexpectedly, our results did not show differences between groups, and this can be interpreted from the limited cohort of patients when divided for BMI.

In the literature, DMC is considered an optimal implant with excellent survivorship, and low dislocation and overall complication rates. Therefore, it can be considered a safe and effective option, particularly in high-risk patients undergoing revision THA for instability [22,23,27]. Stucinskas et al. [18], in a study from the Lithuanian arthroplasty register, compared the re-revision rates of DMC to other surgical constructs when treating recurrent dislocations after revision THA. There were only 2% of re-revisions due to a dislocation in DMC vs. 9% when other constructs were used. A study from the Dutch register by Bloemheuve et al. [19] on 15.922 cup revisions with a 5-year follow up, showed a re-revision rate of 3.5% for DMC (95% CI 3.0–4.2) and 6.7% (CI 6.3–7.2) for standard cups. Another study from the Swedish hip arthroplasty register by Mohaddes et al. [17], showed that at a 4-year follow-up, DMCs compared to standard implants had a lower risk for re-revision for all reasons (91% ± 3.7% vs. 86% ± 4.1%, *p* = 0.02); the significance was even higher when re-operation for dislocation was considered (96% ± 3.0% vs. 92% ± 3.3%, *p* = 0.001). DMC reduced the short- to mid-term risk of a revision after primary implants compared with classic cup designs. Different findings in the literature suggest a better function of DMC implants in unstable THA undergoing revision surgery compared to standard implants at a short-term follow-up. Our study supports these findings at a longer follow-up of 10 years. 

Clinical studies clearly outline how DMC implants perform by evaluating the risk of re-revision due to dislocation or instability. Hernigou et al. [28], in his study showed that the number of dislocations in THAs revised by standard implants was 15.6% (5 of 32) at a one-year follow-up compared with 0% in DMC THA; at five years, the rate was 21.8% (7 of 32) for standard cups compared to 2.8% in DMC (1 of 35). Schmidt et al. [29], in a retrospective study of 295 revision THAs with an average follow-up of 2.3 years, divided into DMCs (184 revisions) or standard cups (111 revisions), showed a risk of dislocation for DMCs of 3.8% (7/184), and with a standard cup of 13.5% (15/111). Similarly, other studies showed how DMCs can be a viable solution to managing THA instability and to achieve a lower dislocation rate [29,30,31,32]. These findings are comparable with the results of the current study, confirming that DMC is a suitable option to reduce the risk of re-revision in patients operated on for THA revision surgery for dislocation. 

Implant survival is a fundamental parameter to promote the use of a determinate implant in clinical practice. Ecker et al. [25], in his study on DMCs used in complex revision THAs, showed survival rates of 96% at 5 years (95% CI: 92 to 98) and 82% at 9 years (95% CI: 72 to 89). The overall dislocation rate was 11% (24 of 216 patients) at the final follow-up, with more revision than expected based on earlier studies on dislocations of these components. Li et al. [33], in a study on 267 cases of revision THA, found that DMCs were used more frequently in patients revised for instability compared with the standard group (8.5% vs. 1.2%, *p* < 0.005), and had a reduced incidence of postoperative dislocations (2.1% vs. 8.7%, *p* = 0.067), and no difference in the rates of re-revisions (9.6% vs. 11.6%, *p* = 0.770). De l’Escalopier et al. [30], in a retrospective study, found that DMCs implanted in revision THAs for dislocation had a survival rate at 7 years of 90.4 ± 5.3%. The good survival and outcomes of DMC implants promoted their use and warranted the extension of the surgical indications [15,23,27]. Our study supports this attitude and show the validity of DMC implants in the management of the recurrent dislocation of THA implants requiring revision surgery, confirming that modern implants are associated with good implant survival. 

Beyond DMC implants, another viable option is the use of standard cups with or without a posterior lip augmentation device, but using a bigger femoral head, to increase the jumping distance and to reduce the risk of dislocation [34]. 

In the literature, different authors explored the topic of femoral head size compared to DMCs, because it is well known that bigger diameter femoral heads reduce the risk of dislocation [35]. Pituckanotai et al. [36] in his systematic review and meta-analysis recommended using DMCs and big head diameters to improve implant stability, even though the study conclusions were limited by the short-term follow-up of 5 years. Sonn et al. [37], in a retrospective study of 301 revision THAs divided according to the use of DMC implants or standard cups with a femoral head of ≥40 mm, found that there were no differences in dislocation rates between the implants. Moreover, Hoskins et al. [38] in a meta-analysis compared primary and revision DMC constructs versus large femoral head bearings in primary and revision THAs; they found a clear advantage for the use of DMCs in primary implants, while in THA revisions, the difference in the risk of revision surgery for dislocation was unclear. They remarked that the topic lacked a literature with medium- to long-term follow-up. The current study fills this gap and suggests that the use of big femoral heads of ≥36 mm or DMC implants in revision THA for instability is beneficial at a longer follow-up.

## 5. Conclusions

In conclusion, DMC or femoral heads of ≥36 mm are a valid choice to manage THA instability, and their use is associated to a reduced revision rate for dislocation in a mid-term follow-up. In those selected and targeted patients, these options should be taken into consideration to improve the outcomes. A longer follow-up and prospective studies may confirm the benefits of these implants in revision THA in reducing instability, improving surgical outcomes, and increasing patient satisfaction.

## Figures and Tables

**Table 1:** Demographic age information divided by gender.

	F (65.6)		M (34.4)		Total	
Age Range	N	%	N	%	N	%
<40	1	0.6	0	0.0	1	0.4
40–49	6	3.6	2	2.3	8	3.2
50–59	10	6.0	9	10.3	19	7.5
60–69	38	22.9	18	20.7	56	22.1
70–79	69	41.6	43	49.4	112	44.3
≥80	42	25.3	15	17.2	57	22.5
Total	166	100.0	87	100.0	253	100.0

**Table 2:** Demographic BMI information.

BMI	N	%
Underweight	2	0.8
Normal weight	61	24.1
Overweight	108	42.7
Obese	42	16.6
Missing	40	15.8
Total	253	100.0

**Table 3:** Implants divided by BMI groups.

	Standard		DMC		Total	
BMI	N	%	N	%	N	%
Underweight	1	50.0	1	50.0	2	100.0
Normal weight	46	75.4	15	24.6	61	100.0
Overweight	89	82.4	19	17.6	108	100.0
Obese	32	76.2	10	23.8	42	100.0
Missing	28	70.0	12	30.0	40	100.0
Total	196	77.5	57	22.5	253	100.0

**Table 4:** Overall survival of Standard Cups and DMC.

	% Survival (95% CI)					
Groups	1 Y	3 Y	5 Y	7 Y	10 Y	15 Y
Standard cup	88.7 (83.4–92.4)	86.5 (80.9–90.6)	85.1 (79.2–89.5)	84.2 (78.0–88.8)	77.9 (69.7–84.3)	74.3 (65.0–81.9)
Implants at risk	169	134	106	81	51	13
DMC	94.5 (84.4–98.2)	90.0 (77.9–95.8)	82.0 (67.7–90.9)	82.0 (67.7–90.9)	77.5 (60.8–88.4)	
Implants at risk	51	39	29	24	15	-

**Table 5:** Summary of re-failure causes.

	Standard Cup			DMC		
Re-Failure Causes	N	IR (%)	Failure Cause	N	IR (%)	Failure Cause
Aseptic global loosening	3	1.5	8.3	1	1.8	11.1
Aseptic cup loosening	4	2.0	11.1	3	5.3	33.3
Aseptic stem loosening	4	2.0	11.1	0	0.0	0.0
Septic loosening	4	2.0	11.1	1	1.8	11.1
Dislocation	11	5.6	30.6	0	0.0	0.0
Periprosthetic fracture	1	0.5	2.8	2	3.5	22.2
Pain without loosening	0	0.0	0.0	1	1.8	11.1
Other	7	3.6	19.4	1	1.8	11.1
Missing	2	1.0	5.6	0	0.0	0.0
Total	36	18.3	100.0	9	15.7	100.0

## Data Availability

Not applicable.

## Abbreviations

BMI	Body Mass Index
DMC	Dual Mobility Cup
ER	Emilia-Romagna
PLAD	posterior lip augmentation device
RIPO	Registro dell’Implantologia Protesica Ortopedica
rTHA	revision Total Hip Arthroplasty
THA	Total Hip Arthroplasty
